# Measuring the harm of sugar sweetened beverages and internalities associated with it

**DOI:** 10.3389/fpubh.2024.1152710

**Published:** 2024-08-27

**Authors:** Ningxin Ding, Jaikishan Desai

**Affiliations:** School of Government, Victoria University of Wellington, Wellington, New Zealand

**Keywords:** sugar sweetened beverages, internalities, contingent valuation, willingness-to-pay, optimal tax rate

## Abstract

**Introduction:**

Obesity, which is partly driven by the consumption of sugar-sweetened beverages (SSBs), significantly increases the risk of type-2 diabetes and cardiovascular diseases, leading to substantial health and economic burdens.

**Methods:**

This study aims to quantify the monetary value of health harms caused by SSB consumption, along with the associated internalities, through a contingent valuation survey. The results are crucial for determining the socially optimal tax rate.

**Results:**

We surveyed 293 residents of Wellington, New Zealand, to assess their willingness to pay (WTP) for reductions in the risks of diabetes, stroke, and heart disease associated with SSB intake. Logistic regression analysis revealed the marginal WTP for a 1% risk reduction in diabetes, stroke, and heart disease to be NZ$404.86, NZ$809.04, and NZ$1,236.84, respectively. Based on these values, we estimate the marginal harm from SSB consumption to be approximately NZ$17.37 per liter in New Zealand, with internalities amounting to NZ$6.43 per liter, suggesting an optimal tax rate of NZ$6.49 per liter.

**Discussion:**

Implementing such a tax is feasible and would likely double or triple the price of SSBs in New Zealand.

## 1 Background

Studies have shown that obesity is a significant risk factor for type-2 diabetes, a chronic disease with severe long-term health (1). In New Zealand, healthcare costs associated with obesity were estimated at NZ$624 million in 2006 (2).

Obesity is a persistent health condition with a complex set of contributing factors, such as genetics, environment, sociocultural influences, and behaviors (3). Among these behavioral factors, unhealthy dietary choices which often involve a substantial intake of calorie-dense foods high in fat and added sugars play a significant role (4). Some public health scholars have argued that a major source of sugar is sugar sweetened beverages (SSBs) (5). SSB is a type of drink that contains added sugars, typically in the form of sucrose or high-fructose corn syrup, and a 12-ounce portion of SSB usually contains 50 g of sugar (6). These added sugars contribute to the beverage’s sweetness and caloric content. Sugar-sweetened beverages include a wide range of products, such as soft drinks, sport drinks, and flavored milk (6). Data has shown SSBs constituted approximately 5% of whole household food expenditure in New Zealand (7). Admittedly, energy is essential to survival and functioning, but it does not have to be sourced from sugar and SSBs. SSBs are high in calories but have little or no nutritional value (8).

The link between SSB consumption and obesity, diabetes, and cardiovascular disease has been identified in many studies (9–13). For example, Ludwig found that an additional unit of SSB consumption per day increases the risk of developing obesity by 60% (9). Notably, Schulze et al. found a substantial weight gain in females with escalating SSB intake (10). Furthermore, various studies indicate that men with light-to-medium SSB consumption face a 7 to 9% higher risk of diabetes and a 2 to 4% elevated risk of heart disease (11–13). Additionally, Eshak et al. found that light-to-medium SSB consumers have a 3 to 12% higher risk of stroke compared to non-drinkers (14). Heavy SSB consumers experience even more significant risks. De Kong et al. highlighted a 24% higher risk of diabetes in intensive SSB drinkers compared to non-SSB drinkers (11). Another study revealed that heavy SSB drinkers face a 13 to 14% higher risk of stroke and a 22% higher risk of heart disease compared to non-drinkers (15). Further details of the selected studies are provided in Appendix Table 1.

The above health harm is not often fully accounted for when people are making consumption decisions (16). Consequently, internalities occur, and SSB consumption in market equilibrium is above the optimal level, which yields dead-weight loss to society. Therefore, various interventions have been proposed to solve the problem. These interventions include, but are not limited to, SSB taxes, health education, social media campaigns, and promotion of physical activities (17). These interventions either try to reduce SSB consumption or increase people’s energy expenditure, thereby achieving weight-loss. Particularly, SSB taxes are favored by many governments and policymakers as the costs of implementation for such taxes are relatively low, and the taxes may be paid by a broad spectrum of the population, thereby generating considerable tax revenues (18). Consequently, SSB taxes have been applied in several countries and areas worldwide (19). For example, 24 states and six cities in America have passed SSB taxes since 2009. Other countries, such as the UK, Mexico, Chile, Finland, Hungary, and Ireland, have all imposed taxes on SSBs (18–20).

Goiana-da-Silva et al. present evidence from various countries, demonstrating that most of these taxes can lead to reduced consumption of unhealthy beverages and subsequent health benefits, supporting their implementation as part of broader public health strategies (21)^.^

Although New Zealand has not yet implemented SSB taxes, Ni Mhurchu et al. argue that a 20% SSB tax could effectively address the high burden of diet-related diseases in the country (8). This aligns with the WHO’s recommendation for at least a 20% SSB tax, which has been adopted by many countries (20). However, there are gaps regarding whether the 20% tax rate, or another tax rate, is at the socially optimal level or not.

According to utilitarianism, actions are justified if they benefit the majority (22). Determining the socially optimal tax rate is crucial, as only at this level is welfare maximized (22). Once the optimal level is determined, we can then assess whether the benefits of taxing SSBs are larger than the harm. Pigou stated that the optimal corrective tax should equal the sum of the internalities and externalities (23). Building on Pigou’s idea, Marron developed a formula for the socially optimal tax rate using a utility maximization model (16). He concluded that the corrective tax should be set to reflect the portion of marginal harm the consumer does not account for (internalities), assuming no externalities are present (16). Given this, measuring the internalities associated with sugar-sweetened beverage (SSB) consumption is crucial for determining the optimal tax rate.

Allcott et al. quantified internalities in terms of consumer bias using the concept of a “counterfactual normative consumer.” After examining nutrition knowledge and excessive SSB consumption across 18,000 households, they found that if individuals had perfect self-control and the knowledge of nutritionists or dietitians, SSB consumption per US household would decrease by 31 to 37 percent (24). However, their study does not account for all societal costs, such as time costs, travel costs, psychological losses of friends and family members due to a patient’s illness, and losses in productivity and income due to illness. This omission suggests that internalities may be underestimated. To address this, one could use contingent valuation (CV) to ask the public about their willingness-to-pay (WTP) for health risk reductions associated with excessive SSB consumption.

Building on the above discussion, the objective of this study is to measure the monetary value of health harm from SSB consumption. We conducted a contingent valuation survey to estimate people’s WTP for health risk reductions. The results were used to calculate the monetary value of harm from SSB consumption, the associated internalities, and the optimal tax rate.

## 2 Methodology

### 2.1 Study design

In this section, we outline the methods used to measure the health harm from sugar-sweetened beverages (SSBs) and the internalities associated with their consumption. We also describe the statistical analyses conducted to evaluate the collected data. The following subsections provide a comprehensive breakdown of these methodologies.

If we assume the marginal health harm of SSB consumption is constant, then it will be equal to the average harm which can be calculated as the total health harm divided by total SSB consumption. Let H_t_, N_ssb_, and H_I_ denote total health harm, total SSB consumption, and the average health harm per liter, respectively. The relationship between the three terms is:


(1) $$H_{I}=\frac{H_{t}}{\mathrm{Nssb}}$$

The total health harm from SSB consumption can be calculated as the total loss from diseases which includes both the decrease in health and other non-health losses (time costs, reduction in productivity, etc.) due to illness, but excludes healthcare expenditures publicly funded that are considered as externalities. For example, if the monetary value of loss from illness is $10,000 for a SSB consumer, and the average SSB consumption for the consumer is 1,000 liters, then the average health harm is calculated as $10,000/1,000 = $10/liter.

As discussed previously, only a proportion of the health harm is acknowledged and considered by consumers. Let $\beta \in \left(0,1\right)$ represent the proportion of health harm considered by consumers, so that $\left(1-\beta \right)H_{I}=\left(1-\beta \right)\frac{H_{t}}{\;\mathrm{Nssb}}$ is internalities (per liter), that is, the portion of health harm consumers do not consider. Studies have shown that if people had perfect self-control and were acknowledged as health professionals, SSB consumption would decrease by 31 to 37% (24). This indicates that 1–β = 0.37, so that *β* = 0.63; and if 1–β = 0.31, so that *β* = 0.69. In the base case, 0.63 is used as the value of the proportion of harm a consumer considered which was later changed to 0.69 in the sensitivity analysis (24).

Apparently, to investigate the value of internalities, N_ssb_ and H_t_ have to be estimated. The next few sections describe the methods used to measure these values.

- Estimating SSB consumption N_ssb_.

The SSB consumption is estimated based on the following information. Statistics indicate that the weekly household expenditure on soft drinks is $7.91 (Food price index: November 2018; available from Statistics NZ) and the average price of SSBs is $2.50 per 1.5 liters.^1^ Given these, the unit price is $2.5/1.5 = $1.67/liter, so the average weekly household consumption is $7.91/$1.67 = 4.75 liters. Since the weighted average number of people per household is 3.50 (25), weekly SSB consumption per person and yearly SSB consumption per person are 4.75/3.50 = 1.356 liters and (1.356/7)*365 = 70.71 liters (N_ssb_ = 70.71 L) respectively.

In order to deal with uncertainty in SSB consumption due to household size, for household size = 8 or > 8, the average number of people per household was changed to 12 (increased by 50%) in the sensitivity analysis. This modification changed the weighted average number of people per household to 3.62, and SSB consumption per person per year decreased to 68.36 liters.

- Measuring total health harm H_t_.

Because SSB consumption is associated with increased diabetes, stroke, and heart disease risk (4, 11–13), we can try to measure H_t_ in a contingent valuation (CV) survey which estimates people’s WTP for health risk reductions, and in this context, losses from diseases are revealed by people’s WTPs. Let WTP denote people’s WTP for a 1% reduction in health risks, and R is the reduction in health risks. Now Equation 1 becomes Equation 2:


(2) $$H_{I}=\frac{H_{t}}{\mathrm{Nssb}}=\frac{{WTP}^{*}R}{\mathrm{Nssb}}$$

For example, compared with a non-drinker, a SSB drinker has a 10% higher risk of developing diabetes, the person’s WTP for a 1% reduction in diabetes is $1,000, and the average SSB consumption for the consumer is 10,000 liters, then the average harm from SSB consumption = $1,000*10/10,000 = $1/liter.

In this base case of this study, we conservatively assume that the increased risk of diabetes, stroke, and heart disease is 7, 3, and 2%, respectively, (details are provided in Appendix Table 1). In order to deal with uncertainty in disease risks, the increased diabetes risk, stroke risk, and heart disease risk were changed to 9, 12, and 4%, respectively, based on the findings of literature summarized in Appendix. The next few sections describe the methods used to measure people’s WTP for health risk reductions in a CV survey.

- Design of the CV survey.

The methodological approach employed in this study utilizes the conjoint or choice experiment approach, originally developed by Green (26). This method is based on Lancaster’s perspective that consumption derives utility from attributes rather than the goods themselves (27). Conjoint analysis is a stated preference method in which respondents make a series of contingent choices based on the attributes presented in the choice set. Our choice set included cost as one attribute and three key health risks we aimed to value. By dividing the attribute coefficient by the cost coefficient, the marginal value of a one-unit change is monetized (28).

The theoretical foundation of random utility stated preference models that underlie the empirical discrete choice models used for estimation begins with an individual’s utility function. To empirically implement this utility framework within a dichotomous choice stated preference survey, we follow Hanemann’s exposition of the utility difference foundation of random utility models. In this model, the first choice is a “no action” or baseline risk level associated with no cost, while the alternative involves reducing the three health risks at a one-time cost of $Z (29). The probability of choosing the action alternative is related to the expected gain from receiving the health risk reduction compared to the price of the hypothetical pill. If this expected utility difference is linear in its arguments and the associated additive random error term is distributed logistically, maximum likelihood statistical routines such as logit models can be used to estimate the following equation (28):


$$\log\left(\frac{P\left(\mathrm{Yes}\right)}{1-P\left(\mathrm{Yes}\right)}\right)=\beta_{0}+\beta_{1}\left(\$Z\right)+\beta_{2}\left(\mathrm{health}\;\mathrm{risk}\;\mathrm{reduction}\right)$$

The marginal value to a person of reducing a health risk of death (or people’s WTP for health risk reduction) is given by $\frac{\beta_{2}}{\beta_{1}}$ (28).

Building on this framework, we designed a survey that posed a series of dichotomous choice questions to participants. These questions aimed to capture their willingness to pay for specific health risk reductions. The following example illustrates the survey design:

**Table:** 

No.	Scenario			Response
	Would you be willing to pay $1,000 at one go for the pill if it were to reduce risk of developing diabetes, stroke, and heart disease by [amounts below] respectively in the next ten years?			
Risk Reduction for…	Diabetes	Stroke	Heart disease	
	7%	3%	2%	Yes/No

As the illustration shown above, dichotomous questions were given to participants. They were asked to make decisions about whether to pay a certain amount of money to reduce the health risks by different levels in the next ten years. The risks of diabetes, heart disease, and stroke assigned to participants were set at the values from the main findings of the literature listed in Appendix Table 1. In order to ensure that the reported WTP values not only include the health harm of diseases, but also account for the loss in productivity and time cost, a sentence was written in the questionnaire to remind respondents that health problems will not only reduce quality of life, but also cause income loss and other costs as well.

In the experiment, the price of the hypothetical pill assigned to participants was stated to be one of eight levels: $1,000, $3,000, $5,000, $10,000, $30,000, $80,000, $180,000, and $382,000 ($382,000 was the highest value seen in our open-ended pilot survey).

As the possible combinations of health risks and the price of the hypothetical pill were too numerous to all be surveyed, the D-optimal fractional factorial algorithm was applied to create the optimal 72 combinations (listed in Appendix) (30). It should be noted that unlike the most conventional approach of presenting one scenario to each respondent and varying WTP values by subsamples, every participant was randomly assigned twenty-seven scenarios, each representing a different combination of health risks and the price of the hypothetical pill. Specifically, each respondent answered only one valuation question during each instance, resulting in a total of 27 responses per participant.

### 2.2 Population and inclusion criteria

Given the suggestions and instructions provided by the Victoria University of Wellington Human Ethics Committee and the limited financial resources available, the CV survey was sent to Facebook users in 56 Wellington community groups. In addition, participants were required to meet the following inclusion criteria: (1) above 16 years old, and (2) had lived in New Zealand for one-year at least. Informed consent was provided at the beginning of the survey, and participants were allowed to stop answering questions at any point of their choosing.

### 2.3 Data collection and selection

As discussed previously, samples were drawn from the 56 Facebook community groups in Wellington, New Zealand, from June 5th to December 7th, 2019. Many Facebook community page administrators only allowed repeat advertising once per week, and some of them only allowed once per month. On some occasions, advertisements were automatically identified as a scam by Facebook. As a result, the number of individuals per community group was quite low, which was approximately 9 participants on average, and 493 in total. However, 114 of them stopped at the information sheet page, and another 74 stopped at, or before the WTP questions page. Since the answers from the people who stopped halfway were not recorded and returned by Qualtrics, they were excluded in analysis. Consequently, a total of 305 responses were returned.

**Figure 1:**
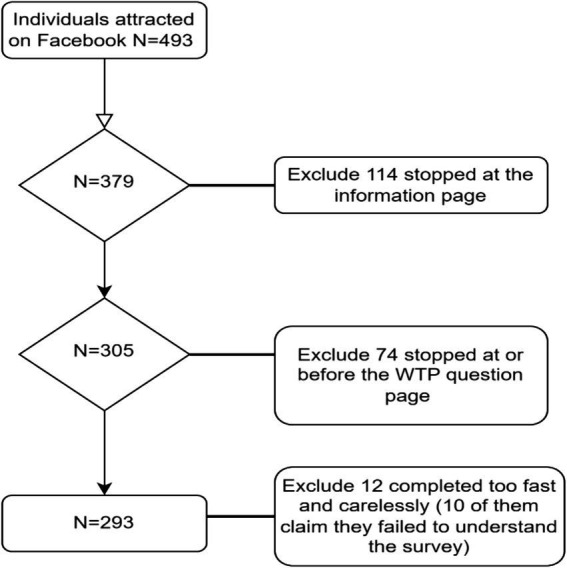
Flowchart of participants entry into the study.

Not all data were included in the analysis, and the selection was based on two criteria: (1) how well the scenarios were understood by the participants, and (2) how much time each individual spent on the survey. There were 10 participants who declared that they failed to understand the scenarios described in the questionnaire, and all of them finished the survey in 2 min. They were excluded from the survey as their answers were thought meaningless. In addition, Qualtrics automatically recorded the time used to finish the survey, which gave an indication of how seriously the survey was treated by each participant. Given the length of the questionnaire and the complexity of the scenarios, it was thought to be impossible to finish the survey in two minutes with serious deliberation about the scenarios described in the questionnaire. Therefore, having excluded 12 participants who completed in 2 min (10 of them declared that they failed to understand the scenarios described in the questionnaire), 293 valid responses were included in analysis. Furthermore, as shown in Appendix Table 2, the sample lacks balance in terms of gender distribution: there were 244 females and 49 males in the sample. Therefore, weights (female: male = 0.63:2.82) were given to address this anomaly (Figure 1).

### 2.4 Study variables and covariates

As discussed earlier, participants were given a questionnaire which asked them to make a series of choices. The marginal value of a one-unit change in each health risk is revealed by the ratio of the attribute coefficient to the cost coefficient, which can be yielded using a binary logistic regression. Specifically, participants’ decisions (Yes/No) were analyzed by the formula below, and the marginal WTP for risk reductions for diabetes, stroke, and heart diseases were estimated as β_diabetes_/β_price_, β_stroke_/β_price_, and β_heart disease_/β_price_, respectively (28):

$\log\left(\frac{P\left(\mathrm{Yes}\right)}{1-P\left(\mathrm{Yes}\right)}\right)=\beta_{0}+\overset{13}{\underset{i=1}{\sum }}\beta_{i}X_{i}$ ,where P(Yes) is the probability of being willing to pay for the hypothetical pill; X_i_ are the individual characteristics surveyed in the questionnaire, these independent variables are: the reduction in diabetes, stroke, and heart disease risk, price of the hypothetical pill, people’s income (participants’ annual income categorized into ranges), age (age of participants categorized into groups), gender, educational level, ethnicity, perceived self-health risk, knowledge of the diseases, the degree to which the scenarios described in the questionnaire are understood, and whether believe public health expenditures should be increased or not. All the independent variables were put into the regression simultaneously.

### 2.5 Statistical analysis

The Wald test was used to test which variables had significant effects on participants’ decisions. Multicollinearity was checked by calculating the Variance Inflation Factor (VIF), to ensure the parsimony of the logistic model. The fitness of the logistic regression was tested with the Cox R-squared value, McFadden R-squared value, and Nagelkerke (Cragg and Uhler) R-squared value. All statistical analyses were conducted using R software, version 4.2.2, and a *p*-value of 0.01 or less was considered statistically significant, unless otherwise specified.

### 2.6 Ethical aspects

To minimize emotional harm to participants, protect their privacy, and ensure compliance with the Treaty of Waitangi, the sampling method, inclusion and exclusion criteria, and analysis plan were approved by the Victoria University of Wellington Human Ethics Committee (0000025927) prior to contacting Facebook community administrators. Permission was obtained from these administrators before the surveys were released online. Participants were informed of their right to withdraw from the study at any time, and all responses were anonymized to maintain confidentiality. Furthermore, the study findings will be disseminated in a way that ensures no individual participant can be identified.

## 3 Results

The survey yielded approximately 106 observations per scenario on average, and 7,632 observations in total. It should be noted that of the 293 selected participants, some of them missed one or two WTP questions due to carelessness.

As discussed previously, logistic regression was applied to investigate people’s decisions and to estimate the marginal WTP for risk reductions. We found that the three health risk reductions, the price of the hypothetical pill, age, perceived self-risks, how well the scenarios described in the questionnaire were understood by participants, race, and the degree to which participants were familiar with the diseases, and educational levels had significant effects (details are shown in Appendix Table 3). Given the coefficients estimated in the logistic regression, the marginal WTP for a 1% reduction in diabetes risk, stroke risk, and heart disease risk are $404.86, $809.04, and $1,236.84, respectively. As shown in Appendix Table 3, the three health risk reductions, the price of the pill, age, perceived self-risks, how well the scenarios described in the questionnaire were understood by participants, race, and the degree to which participants were familiar with the diseases, and educational levels had significant effects at the 0.01 significance level. In particular, a 1% increase in diabetes, stroke, and heart disease risk reductions are predicted to increase the log odds by 0.006, 0.012 and 0.018 respectively, and every additional $1,000 increase in the pill price would lower the log odds by 0.015. Since none of the VIF values are above 10, it might be concluded that multicollinearity is not seen in the regression. The Cox and Snell R-squared, McFadden R-squared, and Nagelkerke (Cragg and Uhler) R-squared are 0.170, 0.230, and 0.300, respectively.

Table 1 shows the estimated monetary value of internalities from SSB consumption. According to the method described previously, given the marginal WTP for diabetes, stroke, and heart disease risk reductions, and the SSB consumption/person/year, the marginal harm a consumer considered is estimated to be $\frac{404.86*7+809.04*3+1236.84*2}{10*70.71}=$ $10.94/liter. When the marginal harm considered ( $\beta H_{I}$ ) is $10.94, and a value of β of 0.63, we can back out the total marginal harm H_I_ is $10.94/0.63 = 17.37/liter. Then the marginal harm that is not considered (internalities) is $6.43/liter, with a possible range of $5.38/liter to $9.90/liter.

**Table 1:** Estimates of internalities.

SSB consumption per person per year	70.71 liters	68.36 liters
Base case disease risks	$6.43/liter	$6.65/liter
Sensitivity analysis disease risks (less conservative)	$9.58/liter	$9.90/liter
Proportion of harm considered (*β* = 0.69)	$5.38/liter	$5.57/liter

## 4 Discussion

This CV study investigated 293 Facebook users in Wellington and found that the marginal WTP for a 1% reduction in diabetes risk, stroke risk and heart disease risk is $404.86, $809.04, and $1,236.84, respectively. Given SSB consumption per person per year, the marginal harm from SSB consumption is approximately NZ$17.37 per liter in New Zealand, the internalities associated with it is NZ$6.43 per liter, and the optimal tax rate is NZ$6.49/liter.

Allcott et al. found that the marginal internality from SSBs is approximately 0.91 to 2.14 cents/ounce on average in the USA (24). However, our study suggested that the harm from internalities is only equivalent to US$0.2/ounce after being adjusted by exchange rate. The substantial difference could be due to the heterogeneities among the people in New Zealand and the USA, in terms of income level and the perceived value of health. In addition, it should also be noted that our estimate in the base case is quite conservative as the harm of dental problems and cancer caused by SSB consumptions were not included. Therefore, there is a risk that the marginal harm of SSB from internalities can be underestimated in this study.

Our results of the contingent valuation survey may also be affected by hypothetical bias. It is not clear whether the amounts stated by participants were their real WTPs or not, as they did not really need to pay money to buy the hypothetical pill. Murphy et al. found that the ratio of hypothetical to actual was 2.6, so the marginal WTPs in the study are also likely to be overestimated due to hypothetical bias (31). Having taken all these factors into consideration, whether the results are overestimated or not cannot be determined.

Furthermore, people’s decisions depended on how severe the participants thought the diseases were, but little detail about the severity of the diseases was provided in the questionnaires. Hence, whether the WTP values revealed the average level of severity or other levels was still impossible to know.

Finally, given that only some Facebook users in the community groups were surveyed in this study, it should be questioned that whether our findings can apply to New Zealand general population. Even if we limit it to Facebook users in Wellington area, the conclusions are still questionable, as there are too many females in the sample.

Despite of the above limitations, one strength of the study is that WTP is a relatively comprehensive measure compared with quality adjusted life years. It includes not only health loss from illness, but also other costs such as time cost, loss in productivity, and emotional harm of relatives and friends.

The validity of the method could be tested by testing whether the association between their WTP values and health benefits are significant or not; and the usual prediction is that people are willing to pay more when benefits are higher (32–34). As discussed previously, the three health risk reductions and price of the pill were significant at the 0.01 significance level. In particular, a 1% increase in diabetes, stroke, and heart disease risk reductions are predicted to increase the log odds by 0.006, 0.012 and 0.018 respectively, and every additional $1,000 increase in the pill price would lower the log odds by 0.015. The results are consistent with the prediction that people are willing to pay more when benefits are higher. In addition, although studies have shown that the reliability of CV method threatened by the lack of understanding of the research questions among participants (32, 33), there is a case for believing that the method is fairly reliable as 88% of the participants claim they were able to well understand the scenarios described in the survey. Given the discussion above, we believe the stated WTP values are probably informative, albeit may be less precise due to the presence of hypothetical bias.

In addition, our findings may have a strong policy implication. Studies have shown that internalities are approximately 100 times greater than externalities (35). This implies that the externalities associated with SSB consumption would be approximately $0.064/liter. According to Pigou, the optimal corrective tax should equal the sum of the internalities and externalities (23). Therefore, when internalities are legitimate concerns, the optimal level of taxes would equal to the sum of internalities and externalities which are approximately NZ$6.49/liter. If a SSB tax of NZ$6.49/liter was imposed, the price of SSBs probably would be doubled or even tripled in New Zealand. Such a high tax rate is thought reasonable and feasible, given that (1) a 100% excise tax has been imposed in Bahrain and Saudi Arabia (18, 19), and (2) many SSB taxes fail to reach their policy goals because consumers fail to notice the price increase caused by the taxes (36, 37). Scholars have found that a SSB tax set at a very low rate (such as 5%) is very likely to be ineffective (36, 37). But when the tax rate increases to 20% or higher, the SSB tax may be able to reach its policy goals (38–41).

Furthermore, evidence indicate that even a 20% *ad valorem* SSB tax is very unlikely to be effective in New Zealand. Bollard et al. conducted an experiment in New Zealand to assess the impacts of a 20% *ad valorem* tax, warning labels, and plain packaging on SSB consumption in 604 young consumers aged 13 to 24 who identified themselves as regular SSB consumers in an online survey in 2014 (42). Participants were randomly allocated to be exposed to one of 12 experimental conditions generated from a computer algorithm. The 12 conditions were combined from an image of branded or plain packaged beverages, with or without a 20% *ad valorem tax,* and with either without any warning, a text warning, or a picture warning. Given one of the specific conditions, participants were asked to show the probability of purchasing using seven-point Likert scales. Their results showed that the decrease in purchase probability associated with a 20% *ad valorem* tax was insignificant (42). The evidence above may provide a justification for a high SSB tax rate in New Zealand.

## 5 Conclusion

In this study, a contingent valuation survey was conducted to estimate people’s willingness-to-pays (WTP) for health risk reductions, the results of which were further used to calculate the monetary value of harm from SSB consumption, the internalities associated with it, and the optimal tax rate. Our estimate shows the marginal harm from SSB consumption is approximately NZ$17.37 per liter in New Zealand, the internalities associated with it is NZ$6.43 per liter, and the optimal tax rate is NZ$6.49/liter. If a tax of NZ$6.49/liter was imposed, the price of SSBs would be doubled or tripled in New Zealand.

## Summary of key points

The marginal harm from consuming sugar-sweetened beverages (SSBs) in New Zealand is estimated at NZ$17.37 per liter. The internalities, representing the proportion of harm not accounted for by consumers, amount to NZ$6.43 per liter. Consequently, the optimal tax rate is calculated to be NZ$6.49 per liter. Implementing this tax would result in the price of SSBs doubling or even tripling in New Zealand.

## Data Availability

The raw data supporting the conclusions of this article will be made available by the authors, without undue reservation.

## Footnotes

^1^ https://figure.nz/chart/WNZOpEoBKRyz4hBh-7p7mC5KFHBt4Rx4u
