# Supplementary material for: Measuring the harm of sugar sweetened beverages and internalities associated with it
Source: Front Public Health. 2024 Aug 27;12:1152710. doi: 10.3389/fpubh.2024.1152710 (PMC11383772; doi:10.3389/fpubh.2024.1152710)
Supplement: Supplementary file 1 [file Table_1.DOCX]

**Appendix**

**Table 1: Summary of Evidence**

| **First Author** | **Risk to Light-to-medium Drinkers Compared with Non-drinkers** | **Risk to Heavy Drinkers Compared with Non-drinkers** |
| --- | --- | --- |
| Bazzano | N/A | 18% higher risk of diabetes |
| Bernstein | N/A | 16% higher risk of stroke |
| De Koning (a) | 7% - 9% higher risk of diabetes for men | 24% higher risk of developing diabetes for men |
| De Koning (b) | 2% - 4% higher risk of developing heart disease | 20% higher risk of developing heart disease for men |
| Eshak | 7% - 12% higher risk of stroke for women; 3% - 12% higher risk of ischemic stroke for women | 21% higher risk of stroke for women; 83% higher risk of ischemic stroke for women |
| Fung | 4% higher risk of heart disease | 23%-35% higher risk of heart disease |
| Huffman | N/A | 19% higher risk of heart disease |
| Larsson | N/A | 19% higher risk of stroke |
| Malik | N/A | 26% higher risks of type 2 diabetes |
| Motonen | N/A | 67% higher risk of diabetes |
| Narain | N/A | 22% higher risk of heart disease, 13% -14% higher risk of stroke |
| Palmer JR | N/A | 24% higher risk of type 2 diabetes |
| Schulze | N/A | One year water drinking (no SSBs) could reduce the risk of type 2 diabetes by 83% |
|  |  |  |

**Table 2: Summary Statistics of Variables and Missing Data for the Survey on Facebook**

| **Variable** | | **No. (%)** | **Missing Data** |
| --- | --- | --- | --- |
| Decision | Yes  No | 5,897 (75%)  1,735 (25%) | 279 |
| Gender | Male  Female | 49 (17%)  244 (83%) | 0 |
| Race | White  Maori  Pasifika  Asian  Other | 184 (63%)  37 (13%)  8 (3%)  43 (14%)  21 (7%) | 0 |
| Understanding | Totally agree  Somewhat agree  Neither agree nor disagree Somewhat disagree | 157 (54%)  99 (34%)  19 (6%)  18 (6%) | 0 |
| High self-risk | Totally agree:  Somewhat agree  Neither agree nor disagree Somewhat disagree  Totally disagree:  Prefer not to say | 46 (16%)  76 (26%)  39 (13%)  88 (30%)  40 (14%)  4 (1%) | 0 |
| More Government Spending | Totally agree  Somewhat agree  Neither agree nor disagree Somewhat disagree  Totally disagree | 126 (43%):  103 (35%):  39 (13%):  16 (5%):  9 (3%) | 0 |
| Educational Level | 2^nd^ education or below  Certi and Dip  Bachelor’s  Postgraduate  Prefer not to say | 57 (19%)  76 (26%)  84 (29%)  61 (21%)  15 (5%) |  |
| Income | Below $25,000  $25,000 to $70,000  $70,000 to $120,000  Above $120,000  Prefer not to say | 57 (19%)  129 (44%)  57 (19%)  19 (6%)  31 (11%) | 0 |
| Good Knowledge of the Diseases | |  |  |
| Totally disagree | | 0 (0%) | 0 |
| Somewhat disagree | | 24 (8%) |  |
| Neither agree or nor disagree | | 24 (8%) |  |
| Somewhat agree | | 134 (46%) |  |
| Totally agree | | 111 (38%) |  |
| Age | |  | 0 |
| Below 25 | | 33 (11%) |  |
| 25 to 45 | | 161 (55%) |  |
| 46 to 65 | | 89 (30%) |  |
| Above 65 | | 8 (3%) |  |
| Prefer not to say | | 2 (1%) |  |

**Table 3: Binary Logistic Regression Analysis on Explain Decisions in the Survey on Facebook**

| **Variable** | **Coefficient (SE)** | **Adjusted Odds Ratios /**  **Exp (β_j_) (95% Confidence Interval)** | **Z value** | **P value** | **VIF** |
| --- | --- | --- | --- | --- | --- |
| Diabetes risk reduction | 0.006 (0.304) | 1.006 (1.004, 1.008) | 6.384 | <0.001*** | 1.025 |
| Stroke risk reduction | 0.012 (0.001) | 1.012 (1.010, 1.014) | 13.926 | <0.001*** | 1.008 |
| Heart disease risk reduction | 0.018 (0.001) | 1.018 (1.014, 1.023) | 7.502 | <0.001*** | 1.025 |
| Income |  |  |  |  | 1.218 |
| 25k to 70k | - 0.208 (0.101) | 0.756 (0.620, 0.922) | - 2.756 | 0.006** |  |
| 70k to 120k | 0.477 (0.118) | 1.611 (1.278, 2.032) | 4.033 | <0.001*** |  |
| Above 120k | - 0.095 (0.161) | 0.909 (0.662, 1.246) | - 0.596 | <0.555 |  |
| Prefer not to say | - 0.733 (0.148) | 0.480 (0.358, 0.641) | - 4.942 | <0.001*** |  |
| WTP | < - 0.001 (< 0.001) | 1.000 (1.000, 1.000) | - 21.581 | <0.001*** | 1.011 |
| Age |  |  |  |  | 1.164 |
| 25 to 45 | - 0.552 (0.119) | 0.576 (0.456, 0.728) | - 4.628 | <0.001*** |  |
| 45 to 65 | - 0.432 (0.132) | 0.649 (0.501, 0.841) | - 3.279 | 0.001** |  |
| Above 65 | 0.604 (0.191) | 1.830 (1.257, 2.661) | 3.159 | 0.002** |  |
| Prefer not to say | - 1.998 (0.970) | 0.136 (0.011, 0.664) | - 2.060 | 0.039* |  |
| Gender |  |  |  |  | 1.222 |
| Male | 0.103 (0.0734) | 1.108 (0.959, 1.279) | 1.397 | 0.163 |  |
| Race |  |  |  |  | 1.181 |
| Asian | 0.749 (0.113) | 2.114 (1.694, 2.639) | 6.619 | <0.001*** |  |
| Pasifika | 0.327 (0.197) | 1.386 (0.936, 2.030) | 1.656 | 0.098 |  |
| Maori | - 0.236 (0.116) | 0.790 (0.629, 0.990) | - 2.032 | 0.042* |  |
| Other | - 0.011 (0.150) | 0.532 (0.394, 0.711) | - 4.200 | <0.001*** |  |
| Understanding |  |  |  |  | 1.275 |
| Totally agree | 0.192 (0.152) | 1.211 (0.900, 1.635) | 1.260 | 0.208 |  |
| Somewhat agree | 0.661 (0.155) | 1.936 (1.433, 2.626) | 4.275 | <0.001*** |  |
| Neither agree nor disagree | - 0.320 (0.206) | 0.726 (0.485, 1.086) | - 1.559 | 0.119 |  |
| Self-risk |  |  |  |  | 1.172 |
| Totally agree | 0.682 (0.129) | 1.977 (1.539, 2.548) | 5.298 | <0.001*** |  |
| Somewhat agree | 0.441 (0.127) | 1.554 (1.213, 1.998) | 3.467 | <0.001*** |  |
| Neither agree nor disagree | 0.545 (0.141) | 1.725 (1.309, 2.276) | 3.861 | <0.001*** |  |
| Somewhat disagree | 0.122 (0.126) | 1.130 (0.883, 1.449) | 0.996 | 0.334 |  |
| Prefer not to say | 0.834 (0.356) | 2.302 (1.141, 4.612) | 2.346 | 0.019* |  |
| Educational Level |  |  |  |  | 1.207 |
| Certificate or Diploma | - 0.041 (0.109) | 0.959 (0.775, 1.189) | - 0.379 | 0.334 |  |
| Bachelor’s | 0.496 (0.105) | 1.642 (1.337, 2.019) | 4.720 | <0.001*** |  |
| Postgraduate | 0.061 (0.121) | 1.063 (0.839, 1.348) | 0.505 | 0.614 |  |
| Prefer not to say | - 0.444 (0.202) | 0.642 (0.430, 0.949) | - 2.196 | 0.028* |  |
| Good Knowledge of the Diseases |  |  |  |  | 1.247 |
| Totally agree | - 0.265 (0.169) | 0.767 (0.552, 1.071) | -1.568 | 0.117 |  |
| Somewhat agree | - 0.565 (0.165) | 0.568 (0.412, 0.786) | -3.431 | 0.001*** |  |
| Neither agree nor disagree | 0.266 (0.183) | 1.304 (0.913, 1.869) | 1.455 | 0.146 |  |
| More Government Spending |  |  |  |  | 1.194 |
| Totally agree | 0.071 (0.225) | 1.074 (0.697, 1.684) | 0.316 | 0.752 |  |
| Somewhat agree | - 0.151 (0.228) | 0.860 (0.554, 1.358) | -0.661 | 0.509 |  |
| Neither agree nor disagree | 0.079 (0.231) | 1.082 (0.694, 1.716) | 0.343 | 0.732 |  |
| Somewhat disagree | - 0.605 (0.273) | 0.546 (0.321, 0.936) | -2.214 | 0.027* |  |
| Intercept | - 1.743 (0.304) | 0.175 (0.096, 0.316) | - 5.725 | < 0.001*** |  |

Significance levels: 0.01‘***’, 0.05‘**’,0.1‘*’; Cox and Snell R-squared: 0.179; McFadden R-squared: 0.230; Nagelkerke (Cragg and Uhler) R-squared: 0.300; Weights (0.627 for females, and 2.824 for males) were used to adjust the distortion in gender distribution in the sample.

**Table 4: Estimates of Internalities**

| **SSB Consumption per Person Per Year** | **70.71 Litres** | **68.36 Litres** |
| --- | --- | --- |
| **Base Case Disease Risks** | $6.43/ litre | $6.65/ litre |
| **Sensitivity Analysis**  **Disease Risks (less conservative)** | $9.58 / litre | $9.90 / litre |
| **Proportion of Harm Considered (β=0.69)** | $5.38 / litre | $5.57 / litre |
